# Regulatory mechanisms of mitochondrial function by cancer-derived exosomes in cachexia

**DOI:** 10.3389/fonc.2026.1715589

**Published:** 2026-05-08

**Authors:** Ryotaro Tomida, Keisuke Ozaki, Yukako Tanaka, Rina Komatsu, Syuya Hirata, Takayuki Uchida, Junya Furukawa, Takeshi Nikawa, Tomoya Fukawa

**Affiliations:** 1 Department of Urology, Institute of Biomedical Sciences, Tokushima University Graduate School, Tokushima, Japan; 2 Department of Nutritional Physiology, Institute of Biomedical Sciences, Tokushima University Graduate School, Tokushima, Japan

**Keywords:** cancer cachexia, exosome, isocitrate dehydrogenase 2, miR-1260b, mitochondrial dysfunction, reactive oxygen species, renal cell carcinoma

## Abstract

**Background:**

Cancer cachexia is a multifactorial syndrome characterized by progressive skeletal muscle wasting and impaired response to conventional nutritional support, affecting up to 80% of advanced cancer patients and contributing to poor prognosis. Excessive fatty acid oxidation and mitochondrial reactive oxygen species (ROS) generation have been implicated in cachexia-associated muscle atrophy, but the underlying mechanisms remain unclear.

**Methods and results:**

We investigated the role of cancer cell–derived exosomes in metabolic alterations of skeletal muscle cells. Exosomes from a pro-cachectic renal carcinoma cell line (RXF393) induced myotube atrophy, enhanced mitochondrial ROS production, impaired mitochondrial respiration, and reduced expression of isocitrate dehydrogenase 2 (IDH2) and respiratory chain complex subunits compared to observations in non-cachectic controls. miRNA profiling identified enrichment of miR-1260b in pro-cachectic exosomes, and transfection with a miR-1260b mimic reproduced these phenotypes, including IDH2 downregulation, impaired antioxidant defense, and mitochondrial dysfunction.

**Conclusion:**

These findings demonstrate that cancer-derived exosomal miR-1260b suppresses IDH2, disrupts mitochondrial redox balance, and promotes muscle wasting. This study reveals a mechanistic link between exosomal miRNAs and mitochondrial dysfunction in cancer cachexia and suggests that maintaining mitochondrial redox homeostasis may represent a novel therapeutic strategy.

## 1 Introduction

Cachexia is a wasting syndrome characterized by persistent loss of skeletal muscle mass that cannot be fully reversed by conventional nutritional support, resulting in progressive functional impairment (1–4). It is commonly associated with chronic inflammatory diseases and represents a multifactorial syndrome. Cancer causes chronic inflammation, and approximately 80% of patients with advanced disease develop cachexia (5). This syndrome markedly reduces body weight, appetite, treatment response, prognosis, and quality of life (QOL) in these patients (6). Furthermore, cachexia directly accounts for approximately 20% of cancer-related deaths (7, 8).

Cancer cachexia differs from cachexia in other diseases because tumor cells act as foreign entities within a chronically inflamed environment. Tumors are characterized by immune cell infiltration, and this process is further accompanied by the secretion of multiple inflammatory cytokines. Additionally, tumor cells secrete hormones and cytokines that act not only at the tumor site but extend systemically to organs such as skeletal muscle, adipose tissue, and the liver (9–11).

Although inflammatory cytokines, such as interleukin-6 (IL-6), have been implicated as key mediators of cancer cachexia, therapeutic strategies targeting single cytokines have shown limited efficacy in reversing muscle wasting or improving clinical outcomes. These observations suggest that inflammation alone does not fully account for the pathogenesis of cancer cachexia and that additional, downstream mechanisms may play critical roles.

In skeletal muscle, a major effect is excessive fatty acid oxidation that drives mitochondrial overproduction of reactive oxygen species (ROS) and leads to subsequent muscle atrophy (12). Our previous study demonstrated that this metabolic shift is accompanied by mitochondrial dysfunction and oxidative stress, identifying aberrant intracellular metabolism as a central feature of cancer cachexia. However, the upstream mechanisms by which tumor-derived signals induce this pathological metabolic reprogramming in skeletal muscle remain unclear.

In this study, we investigated exosomes released by cancer cells as mediators of metabolic alterations in skeletal muscle cells. Exosomes are small extracellular vesicles (40–160 nm in diameter) secreted by cells; they play a critical role in intercellular communication and are implicated in cancer progression and metastasis (13–16). Cancer cells undergo extensive metabolic reprogramming to support rapid growth and progression, and their exosomes are thought to carry molecular information reflecting these changes. Notably, cancer cells secrete more exosomes compared to non-malignant cells (17–20). Because exosomes function as stable carriers of bioactive molecules—including proteins, lipids, and microRNAs—they represent a plausible mechanism by which tumor-derived metabolic signals are systemically delivered to and taken up by distant organs, including skeletal muscle, thereby inducing metabolic changes (21, 22).

The aim of the present study was to elucidate the effects of cancer cell-derived exosomes on skeletal muscle, particularly their impact on muscle cell metabolism, with the long-term goal of developing novel therapies targeting intracellular metabolism in cancer cachexia.

## 2 Materials and methods

Human skeletal muscle myoblasts (HSMM) were obtained from Lonza (Switzerland). The renal cell carcinoma lines RXF393 (cachexia-inducing in xenograft model (12)), A498 (non-cachexia-inducing), and ACHN (IL-6–high, non–weight-loss–inducing (23)) were obtained from the National Cancer Institute (Bethesda, MD, USA) and the American Type Culture Collection (ATCC, USA), respectively.

HSMM cells were maintained in Skeletal Muscle Cell Basal Medium (SkBM-2; LONZA) supplemented with 10% fetal bovine serum (FBS), 0.1% human epidermal growth factor (hEGF), 0.1% dexamethasone, 2% L-glutamine, and 0.1% gentamicin/amphotericin B at 37 °C in a humidified atmosphere containing 5% CO_2_ until 70–80% confluence. Cells were then passaged into 6- and 12-well plates using Accutase (Nacalai Tesque, Kyoto, Japan). After reaching 90–100% confluence, cultures were differentiated into myotubes in Dulbecco’s Modified Eagle’s medium (DMEM; SIGMA) supplemented with 5% horse serum (HS; SIGMA, St. Louis, MO, USA) and 1% penicillin/streptomycin. RXF393 cells were cultured in RPMI-1640 medium (SIGMA) containing 10% FBS, 1% Antibiotic-Antimycotic (Thermo Fisher Scientific, Cleveland, OH, USA), and 1% 200 nmol/L L-glutamine stock solution (Nacalai Tesque) at 37 °C with 5% CO_2_. ACHN and A498 cells were maintained in Eagle’s Minimum Essential Medium (EMEM; SIGMA) supplemented with 10% FBS and 1% Antibiotic-Antimycotic under identical culture conditions.

### 2.1 Exosome isolation

After reaching 80% confluence of each cancer cell line in 150-mm dishes, RXF393 cells were maintained in RPMI-1640 medium (SIGMA) containing 10% exosome-depleted FBS, 1% Antibiotic-Antimycotic, and 1% 200 nmol/L L-glutamine stock solution at 37 °C with 5% CO_2_. ACHN and A498 cells were cultured in EMEM (SIGMA) supplemented with 10% exosome-depleted FBS, 1% Antibiotic-Antimycotic, and 1% 200 nmol/L L-glutamine stock solution under the same conditions. Culture supernatants were collected from each cell line and concentrated at 4 °C by centrifugation at 4000 rpm using Amicon Ultra filters (Millipore, Burlington, MA, USA). This was followed by exosome isolation with the PS affinity method using the MagCapture Exosome Isolation Kit PS (FUJIFILM, Tokyo, Japan). The isolated extracellular vesicles were identified as exosomes by Western blotting using the exosomal markers CD9, and CD81. Immunoblotting was performed using diluted anti-CD9 (1:1000; anti-mouse, # SHI-EXO-M01, Cosmo Bio Co., Ltd. Tokyo, Japan) and anti-CD81 antibodies (1:1000, anti-mouse, #SHI-EXO-M03, Cosmo Bio Co., Ltd). A horseradish peroxidase–conjugated anti-mouse IgG secondary antibody (1:2000; catalog no. 62-6520) was used for detection. Nanoparticle tracking analysis (NTA) using a NanoSight instrument (Malvern Instruments, UK) was outsourced to FUJIFILM Wako for the evaluation of extracellular vesicle size to validate exosomes and for exosome quantification. Additionally, exosome levels were quantified using the PS Capture™ Exosome ELISA Kit (FUJIFILM).

### 2.2 microRNA analysis

Exosomes from RXF393, ACHN, and A498 cells were subjected to miRNA profiling using the 3D-Gene^®^ miRNA Oligo chip. Analysis was outsourced to TORAY (Toray Industries Inc., Tokyo, Japan).

### 2.3 Exosome treatment of HSMM

Differentiated HSMM myotubes were treated with exosomes derived from RXF393 and ACHN cells. Exosomes were diluted to a final concentration of 1.0 × 10¹^0^ particles/mL in DMEM supplemented with 5% HS, 1% Antibiotic-Antimycotic, and 1% 200 nmol/L L-glutamine stock solution, and cells were cultured at 37 °C under 5% CO_2_. Once HSMMs reached terminal differentiation, exosomes isolated from the cancer cell culture supernatants were added and supplementation was repeated every two days.

### 2.4 miRNA transfection

Differentiated HSMM myotubes were co-transfected with either mirVana™ miRNA mimic Negative Control (Ambion, Austin, TX, USA) or hsa-miR-1260b mirVana™ miRNA mimic (Ambion) using Lipofectamine RNAiMAX (Invitrogen, Carlsbad, CA, USA) and employing the forward transfection method according to the manufacturer’s instructions. miRNA transfections were repeated every two days.

### 2.5 Immunostaining

Six days after exosome or miRNA treatment, cells were fixed with 4% paraformaldehyde (FUJIFILM) and permeabilized with phosphate-buffered saline (PBS) containing 0.1% Triton X-100 (Wako Pure Chemical Industries). After blocking, cells were incubated with Anti-Myosin Heavy Chain Mouse Monoclonal Antibody (Funakoshi, Tokyo, Japan) at 3 µg/mL, followed by Goat Anti-Mouse IgG H&L (Alexa Fluor^®^ 488, Abcam) at a 1:1000 dilution. Fluorescent images were captured using a fluorescence microscope (BIOREVO BZ-9000; Osaka, Japan). Fifty myotube diameters per condition were randomly measured and analyzed using ImageJ software (Wayne Rasband, Bethesda, Maryland, USA). Myotube diameter was defined as the maximum width of each individual myotube fiber, measured perpendicular to the longitudinal axis while avoiding tapered ends. For each well, 50 myotubes were measured, and the mean value was calculated and considered as n = 1. Statistical comparisons were performed using n = 3 independent wells per condition.

### 2.6 Measurement of mitochondrial ROS

Ninety-six hours after exosome or miRNA treatment, cells (n=3) were incubated with HBSS/Ca/Mg containing 5 µM MitoSOX Red (Invitrogen) for 20 min. For imaging, the medium was replaced with Hank’s Balanced Salt Solution (HBSS) without antibodies and fluorescence images were captured using a fluorescence microscope. Image analysis was performed using ImageJ software (Wayne Rasband).

### 2.7 Western blotting

Seventy-two hours after exosome or 96h after miRNA treatment, HSMM cells were lysed in Cell Lysis Buffer (Cell Signaling Technology, Danvers, MA, USA). Protein concentrations were quantified using a BCA Protein Assay Kit (Thermo Fisher Scientific), and 20 µg of protein per lane was denatured at 95 °C for 5 min. Proteins were separated on 12% polyacrylamide gels by electrophoresis and transferred to PVDF membranes (Bio-Rad, Hercules, California, USA) using a tank blotting system (Bio-Rad). Membranes were blocked at room temperature for 1 h with 4% Block Ace solution.

Primary antibodies were applied overnight at 4 °C as follows: Anti-Fumarase (D9C5) Rabbit mAb 4567, Anti-IDH2 (D8E3B) Rabbit mAb #56439, Anti-Citrate Synthase (D7V8B) Rabbit mAb #14309, Anti-ACO2 (D6D9) XP^®^ Rabbit mAb #6571, Anti-MPC2 (D4I7G) Rabbit mAb #46141, Anti-MPC1 (D2L9I) Rabbit mAb #14462, Anti-DLST (D22B1) XP^®^ Rabbit mAb #11954, Anti-SDHA (D6J9M) XP^®^ Rabbit mAb #11998, Anti-IDH1 Antibody #3997 (all from Cell Signaling Technology, 1:1000 dilution), Total OXPHOS Rodent WB Antibody Cocktail (Abcam, Cambridge, MA, USA, 1:1000 dilution), and Anti-NDUFS1 (Santa Cruz Biotechnology, 1:100 dilution). Internal control proteins included Anti-TOM20 (Proteintech, Rosemont, USA) and β-actin (Sigma), both at 1:1000 dilution.

Secondary antibodies were applied at room temperature for 1 h using Anti-Rabbit IgG (Cell Signaling Technology, 1:1000) or Anti-Mouse IgG (Invitrogen, 1:2000). Protein signals were visualized using ECL reagents (Cytiva, Marlborough, MA, USA), captured with the ImageQuant LAS 500 system (Cytiva), and analyzed with Image Studio Digits v5.2 (LI-COR). When necessary, after detection of the target proteins, the same PVDF membranes were treated with WB Stripping Solution Strong (Nacalai Tesque) for 15 min at room temperature to remove bound antibodies. The membranes were then re-blocked with blocking reagent and reprobed using a different primary antibody. All Western blot experiments were independently repeated at least three times, and representative images are shown unless otherwise indicated.

### 2.8 Mitochondrial function assay

Seventy-two hours after exosome or miRNA treatment, HSMM cells (n=3) were incubated for 60 min at 37 °C under CO_2_-free conditions in XF DMEM Medium (Agilent Technologies, Santa Clara, CA, USA) supplemented with 10 mM glucose, 1 mM pyruvate, and 2 mM glutamine (all from Agilent Technologies). The oxygen consumption rate (OCR) was measured using a Seahorse XFe24 Flux Analyzer (Agilent Technologies). Sequential injections of 1.5 µM oligomycin, 2.0 µM FCCP, and 0.5 µM rotenone/0.5 µM antimycin (all from Agilent Technologies) were performed at 24-min intervals. The basal respiration, ATP production, maximal respiration, and proton leak were calculated according to the manufacturer’s protocol.

### 2.9 NADP/NADPH measurement

At 144 h after treatment with either the miRNA negative control or the miR-1260b mimic, total NADP^+^/NADPH and NADPH levels in the collected HSMM cells (n=3) were quantified using an NADP/NADPH Assay Kit-WST (Dojin Chemical Laboratory, Kumamoto, Japan). Standards and samples (for total NADP^+^/NADPH and NADPH measurements from the negative control and miR-1260b-treated cells) were prepared in a 96-well plate and absorbance was measured at 450 nm using a microplate reader. Concentrations were calculated from a standard curve. NADP^+^ levels were determined by subtracting NADPH values from total NADP^+^/NADPH.

### 2.10 GSH/GSSG measurement

At 144 h after treatment with either the miRNA negative control or the miR-1260b mimic, total glutathione and oxidized glutathione (GSSG) levels in the collected cells (n=3) were quantified using a GSSG/GSH Quantification Kit (Dojin Chemical Laboratory, Kumamoto, Japan). Standards and samples (for total glutathione and GSSG measurements from the negative control and miR-1260b-treated cells) were prepared in a 96-well plate and absorbance was measured at 415 nm using a microplate reader. Concentrations were calculated from a standard curve. Reduced glutathione (GSH) levels were determined by subtracting GSSG values from total glutathione.

### 2.11 Malondialdehyde measurement

At 144 h after treatment with either the miRNA negative control or the miR-1260b mimic, intracellular MDA levels in the collected HSMM cells (n=3) were quantified using an MDA Assay Kit (Dojin Chemical Laboratory, Kumamoto, Japan). Standards and samples (negative control and miR-1260b-treated cells) were prepared in black 96-well plates and fluorescence intensity was measured at an excitation wavelength of 540 nm and an emission wavelength of 590 nm using a microplate reader. Concentrations were calculated from a standard curve.

### 2.12 Statistical analysis

All data are expressed as mean ± standard error of the mean (SEM). Statistical analysis was conducted using Student’s t-test with a two-tailed test. A p-value < 0.05 was considered statistically significant. Statistical analyses were conducted using Microsoft Excel.

## 3 Results

### 3.1 Effects of cancer-derived exosomes on skeletal muscle cells

#### 3.1.1 Changes in muscle fiber diameter and mitochondrial ROS production

**Figure 1:**
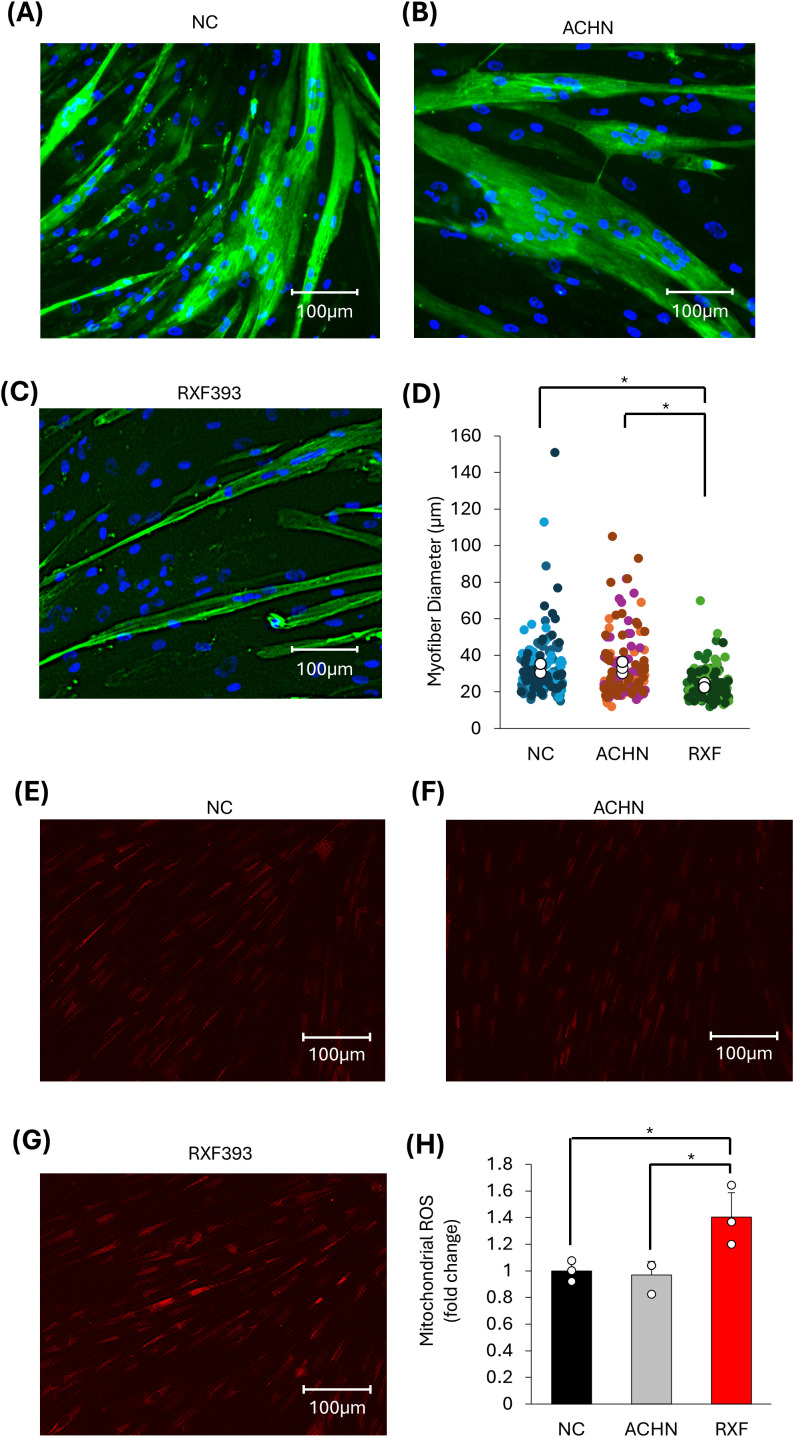
Effects of cancer-derived exosomes on skeletal muscle cells in muscle fiber diameter and mitochondrial ROS production. **(A–D)** Changes in muscle fiber diameter (n=3). **(E–H)** Changes in mitochondrial ROS production (n=3). Scale bar = 100μm; *p < 0.05 by Student’s t-test.

To examine the effects of cancer-derived exosomes on myotubes, fully differentiated HSMM cells were treated with exosomes isolated from RXF393 and ACHN cancer cells. Western blotting confirmed the expression of the exosomal markers CD9 and CD81 in the isolated extracellular vesicles. Nanoparticle tracking analysis (NTA; Nanosight, Malvern Instruments, UK) demonstrated that the vesicles exhibited a size distribution consistent with exosomes. (**Supplementary Figure 1**). Compared with ACHN-derived exosomes, RXF393-derived exosomes significantly reduced muscle fiber diameter (**Figures 1A–D**). Based on these results and previous reports of mitochondrial ROS generation in cancer cachexia models (3), we assessed mitochondrial ROS levels in HSMM cells using MitoSOX Red. Immunostaining revealed elevated mitochondrial ROS in HSMM treated with RXF393-derived exosomes (**Figures 1E–H**), consistent with earlier reports (3). These results indicate that exosomes derived from pro-cachectic cancer cells induce mitochondrial ROS production. In contrast, ACHN-derived exosomes did not induce a significant reduction in myotube diameter nor an increase in mitochondrial ROS production.

#### 3.1.2 Mitochondrial metabolic changes

**Figure 2:**
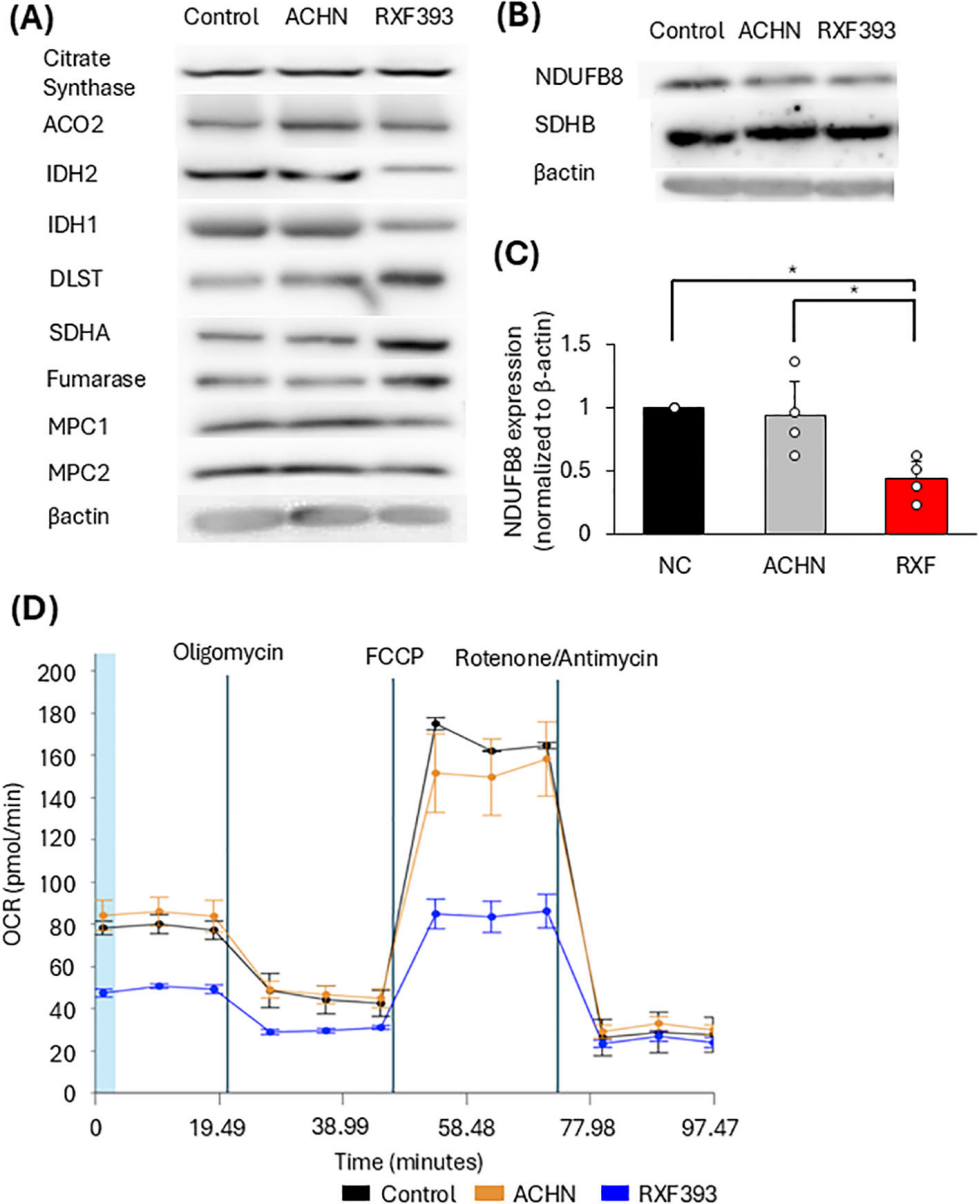
Effects of cancer-derived exosomes on skeletal muscle cells in mitochondrial metabolic changes. **(A)** Western Blot for expression of key enzymes in the TCA cycle. **(B)** Western blot analysis of selected OXPHOS proteins, including Complex I (NDUFB8) and Complex II (SDHA). **(C)** Quantification of NDUFB8 expression normalized to β-actin. **(D)** Mitochondrial respiration was assessed using a flux analyzer after 96 h of cancer cell–derived exosome exposure. (n=3).

To investigate how pro-cachectic cancer cell-derived exosomes increase mitochondrial ROS, we analyzed mitochondrial metabolic alterations. The expression of key tricarboxylic acid (TCA) cycle enzymes and oxidative phosphorylation (OXPHOS)-related proteins was examined. HSMM cells treated with RXF393-derived exosomes exhibited reduced expression of isocitrate dehydrogenase 2 (IDH2), Complex I component (NDUFB8), compared to those in ACHN-derived exosomes-treated cells (**Figures 2A–C**; **Supplementary Figures 1**, **S2**). These results indicate that cancer-derived exosomes disrupt TCA cycle and OXPHOS-related proteins, altering mitochondrial energy metabolism.

#### 3.1.3 Mitochondrial respiration assessed by flux analyzer

Basal and maximal respiration was measured in myotubes treated with cancer-derived exosomes using a flux analyzer to assess the effects of cancer-derived exosomes on mitochondrial function. Both basal and maximal respiration were reduced in HSMM cells treated with RXF393-derived exosomes compared to those of ACHN-derived exosomes (**Figure 2D**). These findings suggest that cancer-derived exosomes impair mitochondrial function, potentially contributing to muscle atrophy.

### 3.2 miRNA analysis in cancer-derived exosomes

To identify molecules within pro-cachectic cancer cell-derived exosomes that may contribute to muscle atrophy, we performed miRNA profiling. RXF393-derived exosomes exhibited a greater than fivefold-increase in specific miRNA expression, including miR-4286, miR-4454, miR-6765-3p, miR-5100, miR-1260b, miR-6126, miR-7975, miR-4497, miR-7977, miR-4745-5p, miR-375-5p, miR-7704, miR-1237-5p, miR-1260a, and miR-6869-5p, compared to that of ACHN-derived exosomes (**Table 1**). Among these, we focused on miR-1260b, which is complementary to IDH2 mRNA and has been linked to renal cell carcinoma prognosis (24).

**Table 1:** Differentially expressed microRNAs in cancer cell lines.

miRNA ID	Log2 (75th percentile normalization)			Ratio (RXF393/ACHN)
	A498	ACHN	RXF393	
hsa-miR-4286	3.036	3.629	6.866	9.429
hsa-miR-4454	7.472	7.575	10.750	9.031
hsa-miR-6765-3p	2.573	3.861	6.950	8.506
hsa-miR-5100	5.850	6.187	9.151	7.804
hsa-miR-1260b	5.621	6.085	8.962	7.350
hsa-miR-6126	4.651	3.336	6.201	7.285
hsa-miR-7975	6.505	6.231	9.011	6.869
hsa-miR-4497	4.120	4.223	6.833	6.101
hsa-miR-7977	6.613	6.827	9.404	5.968
hsa-miR-4745-5p	3.856	3.883	6.460	5.967
hsa-miR-375-5p	4.878	4.563	7.040	5.569
hsa-miR-7704	5.050	4.177	6.587	5.317
hsa-miR-1237-5p	4.317	3.501	5.881	5.205
hsa-miR-1260a	4.410	4.766	7.135	5.164
hsa-miR-6869-5p	3.410	2.836	5.186	5.097
hsa-miR-6089	6.072	5.982	8.289	4.949
hsa-miR-187-5p	2.567	2.774	4.980	4.612
hsa-miR-1908-5p	4.549	3.668	5.830	4.477
hsa-miR-10400-5p	5.475	5.051	7.187	4.393
hsa-miR-3960	6.098	5.651	7.683	4.090
hsa-miR-10400-3p	3.263	2.801	4.830	4.081
hsa-miR-4787-5p	5.061	4.552	6.488	3.827
hsa-miR-4508	4.202	3.946	5.875	3.807
hsa-miR-4488	4.971	4.367	6.279	3.764
hsa-miR-4466	4.824	3.964	5.871	3.749

#### 3.2.1 Effects of miR-1260b on skeletal muscle cells

**Figure 3:**
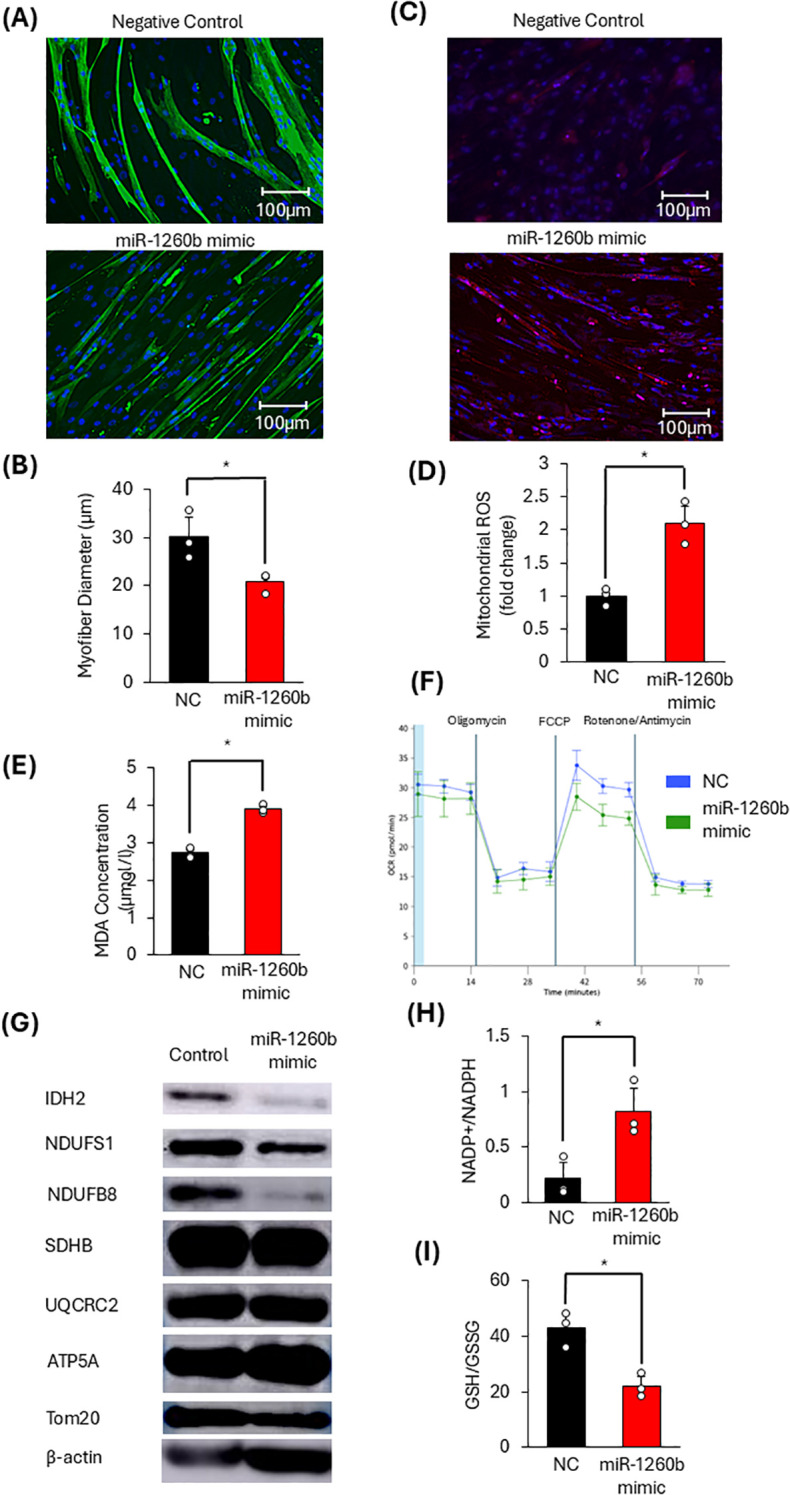
Effects of miR-1260b on skeletal muscle cells. **(A, B)** Changes in muscle fiber diameter (n=3). **(C, D)** Changes in mitochondrial ROS production (n=3). **(E)** MDA Concentration in HSMM cells (n=3). **(F)** Mitochondrial respiration was assessed using a flux analyzer 96 h after miRNA transfection. **(G)**Western Blot for expression of IDH2 and OXPHOS-related proteins. **(H)** NADP^+^/NADPH ratio in HSMM cells (n=3). **(I)** GSH/GSSG concentration ratio in HSMM cells (n=3). Scale bar = 100µm; *p < 0.05 by Student’s t-test.

To examine the effects of miR-1260b on myotubes, HSMM cells were transfected with miR-1260b mimic. To determine an appropriate concentration of miRNA, HSMMs were treated with miR-1260b mimic or a miRNA mimic negative control at concentrations of 10 nM, 20 nM, and 50 nM, and the myotube diameter was evaluated. Treatment with 50 nM miR-1260b mimic resulted in the most pronounced reduction in myotube diameter, whereas no change in myotube diameter was observed in cells treated with the negative control. Based on these findings, a concentration of 50 nM was selected for subsequent experiments using both the miR-1260b mimic and the miRNA mimic negative control (**Supplementary Figure 4**). Transfection induced muscle fiber atrophy and increased mitochondrial ROS production, similar to the effects observed with cancer-derived exosome treatment (**Figures 3A–D**). Additionally, MDA levels were higher in miR-1260b-transfected cells than they were in negative controls (**Figure 3E**). Furthermore, mitochondrial function was assessed in myotubes transfected with miR-1260b using a flux analyzer. In HSMMs transfected with miR-1260b, maximal respiratory capacity was significantly reduced (**Figure 3F**). These findings suggest that miR-1260b contained in cancer-derived exosomes promotes mitochondrial ROS production and induces mitochondrial dysfunction, thereby contributing to muscle atrophy.

#### 3.2.2 Effects of miR-1260b on antioxidant mechanisms

To determine whether miR-1260b reduces IDH2 expression, western blotting was performed at 72 h after miRNA transfection. The miR-1260b mimic reduced IDH2 expression (**Figure 3G**; **Supplementary Figure 5**). To further assess its impact on antioxidant mechanisms, the NADP^+^/NADPH and GSH/GSSG concentration ratios were measured 72 h post-transfection. Reduced IDH2 expression was correlated with an increased NADP^+^/NADPH ratio and a decreased GSH/GSSG ratio, indicating impaired antioxidant capacity (**Figures 3H, I**). These results indicate that miR-1260b transfection is associated with reduced IDH2 expression, accompanied by alterations in cellular redox parameters, including NADP^+^/NADPH and GSH/GSSG ratios.

#### 3.2.3 Effects of miR-1260b on respiratory chain complexes

Given the decrease in OXPHOS function following exosome treatment, we examined the impact of miR-1260b on OXPHOS -related protein expression. HSMM cells transfected with miR-1260b mimic exhibited reduced expression of Complex I components (NDUFS1 and NDUFB8), mirroring the effects of pro-cachectic cancer cell-derived exosome treatment (**Figure 3G**; **Supplementary Figure 5**). These results suggest that miR-1260b, similar to cancer-derived exosomes, affects TCA cycle enzymes and OXPHOS -related proteins, leading to mitochondrial metabolic alterations associated with muscle atrophy.

## 4 Discussion

Cancer cachexia, an inflammatory condition associated with cancer, enhances lipid and protein catabolism, resulting in the loss of adipose tissue (25–27). These metabolic alterations occur early in cachexia development. Excessive fatty acid β-oxidation has been observed in skeletal muscle cells at early stages of cancer cachexia (12). Normally, fatty acids entering mitochondria via β-oxidation are used for energy production; however, in cachexia models, this process generates oxidative stress and contributes to muscle atrophy (12). These findings suggest that mitochondrial metabolic changes occur in skeletal muscle cells during cachexia.

Given that inflammatory cytokines alone do not fully explain cachexia-associated metabolic alterations (28, 29), we focused on tumor-derived exosomes as additional mediators of mitochondrial dysfunction in skeletal muscle. ACHN is known to express high levels of IL-6 (30), one of the most extensively studied cytokines implicated in cancer cachexia. However, despite elevated IL-6 expression, ACHN-bearing xenograft models do not exhibit significant body weight loss, as previously reported (31).

These observations are consistent with our previous findings and those of others, indicating that inflammatory cytokines alone are insufficient to fully account for the development of cancer cachexia or to induce overt cachexia. Rather, additional tumor-derived factors are required to induce sustained metabolic dysregulation in skeletal muscle. As such, ACHN serves as an appropriate control model in which high IL-6 expression is dissociated from systemic wasting, enabling the evaluation of cytokine-independent mechanisms such as exosome-mediated muscle atrophy.

In this context, ACHN represents a biologically relevant control, as it possesses inflammatory characteristics associated with cachexia but lacks robust cachexia-inducing capacity. As demonstrated in our Results section, ACHN-derived exosomes did not induce myotube atrophy or mitochondrial ROS production. This property makes ACHN-derived exosomes particularly useful for distinguishing cachexia-specific metabolic signals from inflammation-associated but non-cachectic signals.

In this study, when exosomes collected from a pro-cachectic renal cancer cell line were added to skeletal muscle cells, ROS was produced in the mitochondria of muscle cells, leading to muscle atrophy. These results are consistent with previous reports focusing on colorectal and lung cancer, in which cancer cell–derived extracellular vesicles were demonstrated to induce muscle atrophy in cancer cachexia (32, 33). Based on these results, we examined the expression of molecules related to the TCA cycle and OXPHOS to clarify the metabolic changes within mitochondria. The results revealed a decrease in the expression of IDH2, a key enzyme in the TCA cycle, and a decrease in the expression of complex I component of the electron transport chain in OXPHOS. These findings confirmed that exosome-induced metabolic changes occur in the mitochondria of skeletal muscle cells. These results are consistent with previous reports demonstrating that extracellular vesicles derived from breast cancer cells induce skeletal muscle mitochondrial dysfunction in cancer (34). Further investigations were then conducted examining the generation of ROS. These results are consistent with previous reports on colorectal and lung cancers, where cancer cell–derived extracellular vesicles induced muscle atrophy in cachexia models (32, 33). To clarify mitochondrial metabolic changes, we examined the expression of TCA cycle enzymes and OXPHOS-related proteins. RXF393-derived exosome treatment reduced the expression of IDH2, a key TCA cycle enzyme, and decreased the levels of Complex I component of the electron transport chain in OXPHOS. These findings confirmed that cancer-derived exosomes induce mitochondrial metabolic alterations in skeletal muscle cells, prompting further investigation of ROS generation.

Exosomes carry biological molecules, including nucleic acids and peptides, from their parent cells. The molecules contained within these cancer cell–derived exosomes may contribute to various biological processes underlying cancer cachexia. Notably, cancer-derived exosomes can induce muscle atrophy via miRNAs (32, 35). miRNA profiling identified those with elevated expression in pro-cachectic cell lines. Among these, miR-1260b (which demonstrated a fivefold increase in expression), was selected for functional analysis. It has been reported as a poor prognostic factor in malignant tumors and was predicted *in silico* to target IDH2 (24, 36, 37). Transfection of miR-1260b into skeletal muscle cells recapitulated the effects of exosome treatment, causing decreased IDH2 expression, increased mitochondrial ROS, and muscle atrophy.

IDH2 is a mitochondrial enzyme that generates NADPH during the conversion of isocitrate to α-ketoglutarate. NADPH serves as a cofactor for the reduction of GSSG to GSH, thereby maintaining redox homeostasis. Thus, IDH2 plays a crucial role in regulating the mitochondrial redox balance and preventing oxidative stress-induced cellular damage (38). In this study, miR-1260b transfection was associated with reduced IDH2 expression, which may have contributed, at least in part, to the observed alterations in the NADP^+^/NADPH balance and GSH/GSSG ratio. Consistent with this notion, miR-1260b transfection was accompanied by increased mitochondrial ROS production and muscle fiber atrophy, suggesting that disruption of redox homeostasis is functionally linked to the development of muscle wasting.

IDH2 expression also influences the regulation of electron transport chain complexes (39). Consistent with earlier observations, cancer cell-derived exosomes decreased IDH2 expression and impaired mitochondrial respiratory chain complex function. To further assess this effect, we examined the impact of miR-1260b mimic transfection. Similar to exosome treatment, miR-1260b mimic transfection reduced the expression of Complex I subunits NDUFS1 and NDUFB8. Notably, NDUFS1, located in the N-module of Complex I, was markedly decreased. These results suggest that miR-1260b–mediated suppression of IDH2 contributes to the reduced expression of Complex I, and this may underlie the respiratory chain dysfunction observed in exosome-treated models.

In contrast, studies using IDH2 knockout Drosophila models demonstrated that the expression of UQCRC2 was not significantly altered before or after IDH2 deletion (39). Moreover, other subunits of the electron transport chain were upregulated in IDH2 knockout Drosophila. Together with our findings, these observations suggest that decreased IDH2 expression may contribute, at least in part, to the impaired respiratory chain function observed following miR-1260b mimic transfection. These observations suggest that decreased IDH2 expression may represent one of several factors associated with mitochondrial respiratory chain impairment following miR-1260b transfection. However, given that multiple mitochondrial alterations were observed, the present data do not allow a definitive conclusion regarding the causal role of IDH2 alone.

Furthermore, consistent results have been reported in other tissues, where IDH2 deficiency in brown adipose tissue was found to lead to oxidative stress, decreased NADPH production, and impaired OXPHOS activity, highlighting the critical role of IDH2 in maintaining mitochondrial function across different biological contexts (40).

Dysfunction of Complex I can lead to mitochondrial diseases, neurodegenerative disorders, and impaired energy metabolism by reducing ATP production and limiting energy supply. Furthermore, respiratory chain impairment generates excessive ROS, leading to cellular damage (41). Consistent with the observed reduction in Complex I expression following pro-cachectic cancer cell-derived exosome treatment, flux analysis revealed reductions in both basal and maximal respiration. As mitochondrial dysfunction is linked to skeletal muscle atrophy (42), these results support the hypothesis that pro-cachectic cancer cell–derived exosomes promote muscle atrophy by impairing respiratory chain complexes.

**Figure 4:**
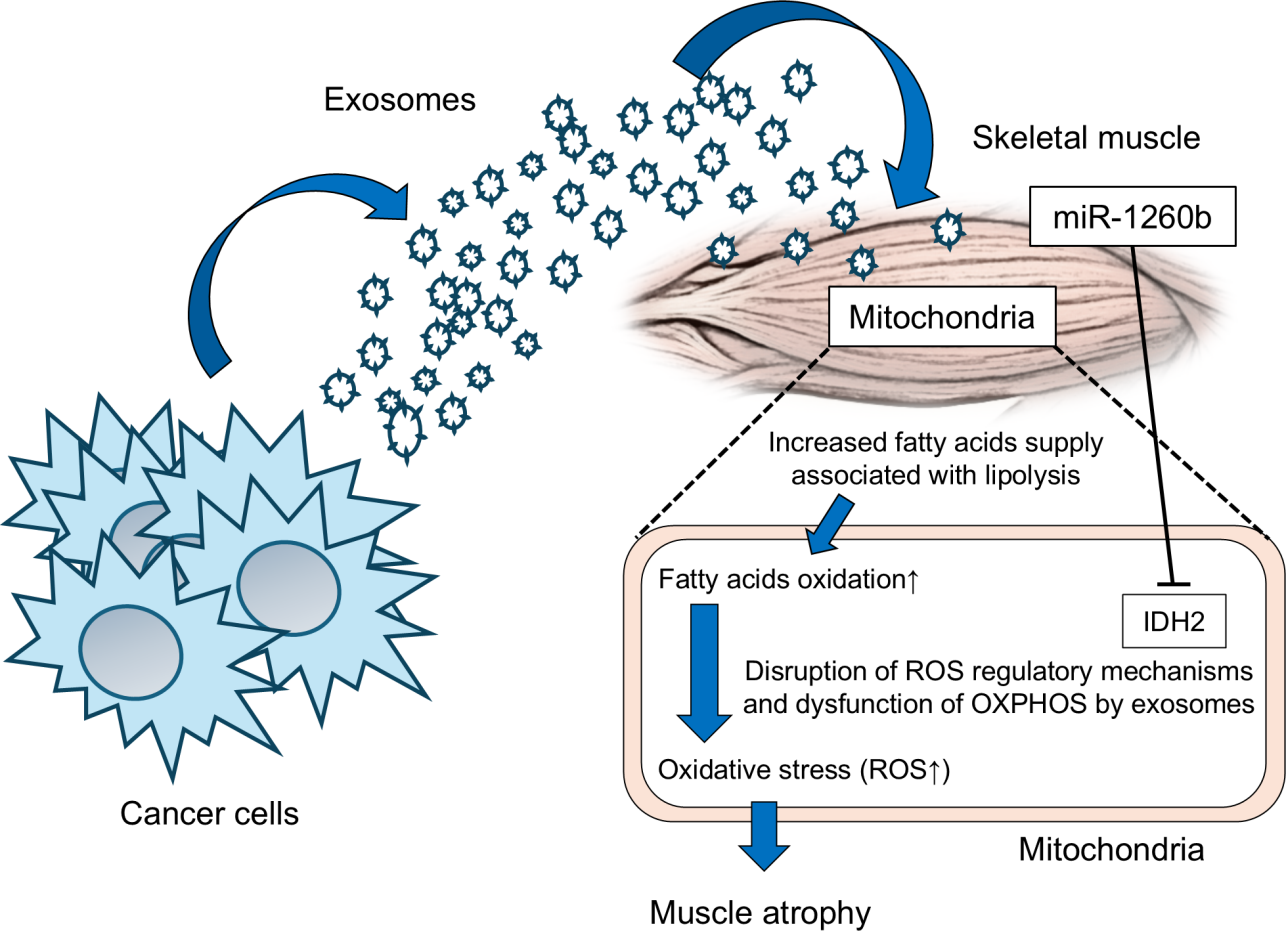
Proposed model of exosome-mediated metabolic alterations in skeletal muscle during cancer cachexia. Tumor-derived exosomes enriched in miR-1260b are taken up by skeletal muscle cells and suppress IDH2 expression, leading to impaired mitochondrial redox balance, increased ROS production, and reduced maximal respiratory capacity. These mitochondrial alterations contribute to skeletal muscle atrophy. Based on previous reports demonstrating excessive fatty acid oxidation in cancer cachexia (12), dysregulated lipid metabolism may further exacerbate mitochondrial oxidative stress. Although lipid metabolic flux was not directly assessed in this study, the schematic integrates the present findings with previously reported metabolic alterations in cancer cachexia.

In cancer cachexia, decreased IDH2 expression caused by cancer cell-derived exosomes is associated with impaired ROS detoxification, excessive ROS production from Complex I dysfunction, and subsequent muscle atrophy. A similar phenomenon was observed with miR-1260b, suggesting its involvement in muscle wasting. These results suggest that miR-1260b within pro-cachectic cancer cell–derived exosomes promotes muscle atrophy by simultaneously increasing ROS production and decreasing ROS-scavenging capacity through downregulation of IDH2 (**Figure 4**).

This study possesses several limitations. First, because only a limited number of cancer cell lines reproducibly induce cachexia-associated phenotypes, this study focused on a single pro-cachectic cell line for mechanistic analyses. Second, our experiments were conducted *in vitro*; as such, it remains unclear whether miR-1260b induces muscle atrophy and mitochondrial dysfunction *in vivo*, and future *in vivo* studies using animal models are required to confirm these effects. Third, although this study focused on miR-1260b and primarily analyzed its functional effects, cancer cell–derived exosomes contain multiple miRNAs and other molecules that may act synergistically to induce muscle atrophy. In addition, miRNAs that were not altered in pro-cachectic exosomes were not functionally tested, and therefore the specificity of miR-1260b relative to other exosomal components warrants further investigation. A comprehensive analysis of additional target molecules other than IDH2 has not yet been conducted. Future studies should therefore investigate the effects of other exosomal components, perform functional inhibition experiments, and systematically analyze target molecules.

## 5 Conclusion

In conclusion, cancer cell–derived exosomes disrupt the TCA cycle, impair respiratory chain complex function, and exacerbate oxidative stress through mitochondrial redox imbalance, ultimately leading to muscle atrophy. These findings highlight the potential contribution of previously reported lipid metabolic reprogramming in cancer-associated muscle atrophy and suggest that therapies that regulate ROS by modulating lipid metabolism may represent therapeutic strategies for treating cancer cachexia.

## Data Availability

The original contributions presented in the study are included in the article and **Supplementary Material**; further inquiries can be directed to the corresponding author.

## Supplementary material

Supplementary Figure 1 Characterization of cancer cell–derived exosomes by NTA and surface marker analysis. Nanoparticle tracking analysis (NTA) was performed to assess the size distribution of extracellular vesicles derived from ACHN and RXF393 cells **(A, B)**. The presence of exosomal surface markers was confirmed by the detection of CD9 and CD81 **(C)**, verifying the exosomal nature of the isolated vesicles. Surface marker analysis was performed for validation purposes.

Supplementary Figure 2 Raw Western blot images correspond to **Figure 2A**.

Supplementary Figure 3 Raw Western blot images correspond to **Figure 2B**.

Supplementary Figure 4 Effects of miR-1260b mimic on myotube diameter in skeletal muscle cells. Skeletal muscle cells were transfected with miR-1260b mimic or negative control (NC) at the indicated concentrations (10, 20, and 50 nM). Representative images are shown for NC **(A–C)** and miR-1260b mimic **(D–F)**. Quantification of myotube diameter is shown in **(G)**. Data are presented as mean ± SEM (n = 3 independent experiments). Scale bar = 50 μm; **p* < 0.05 by Student’s t-test.S

Supplementary Figure 5 Raw Western blot images correspond to **Figure 3G**.
