# Supplementary figures and images for: Regulatory mechanisms of mitochondrial function by cancer-derived exosomes in cachexia
Source: Front Oncol. 2026 May 8;16:1715589. doi: 10.3389/fonc.2026.1715589 (PMC13194054; doi:10.3389/fonc.2026.1715589)

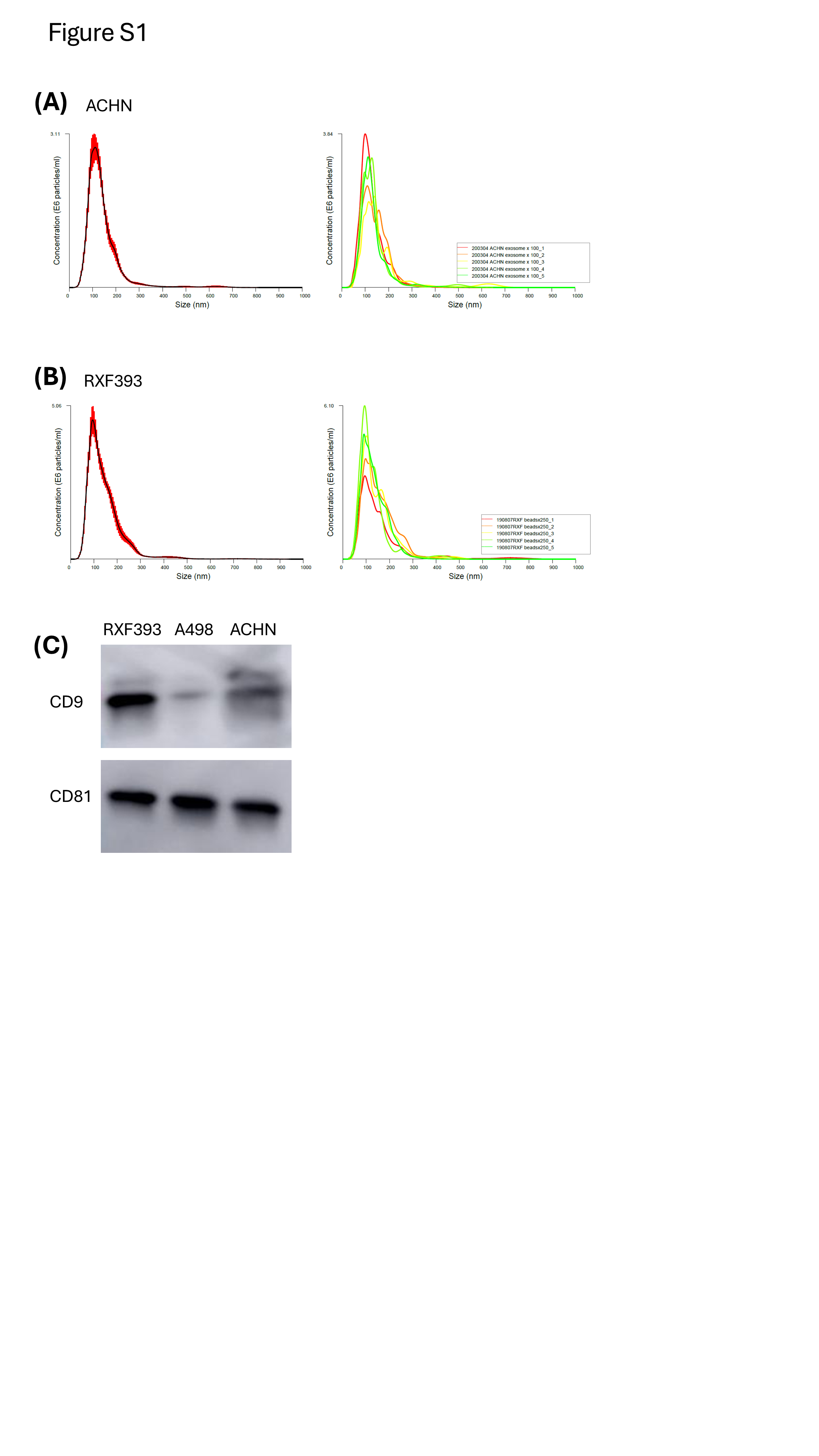

Supplement: Supplementary Figure 1 — Characterization of cancer cell–derived exosomes by NTA and surface marker analysis. Nanoparticle tracking analysis (NTA) was performed to assess the size distribution of extracellular vesicles derived from ACHN and RXF393 cells (A, B). The presence of exosomal surface markers was confirmed by the detection of CD9 and CD81 (C), verifying the exosomal nature of the isolated vesicles. Surface marker analysis was performed for validation purposes. [file Image1.tif]

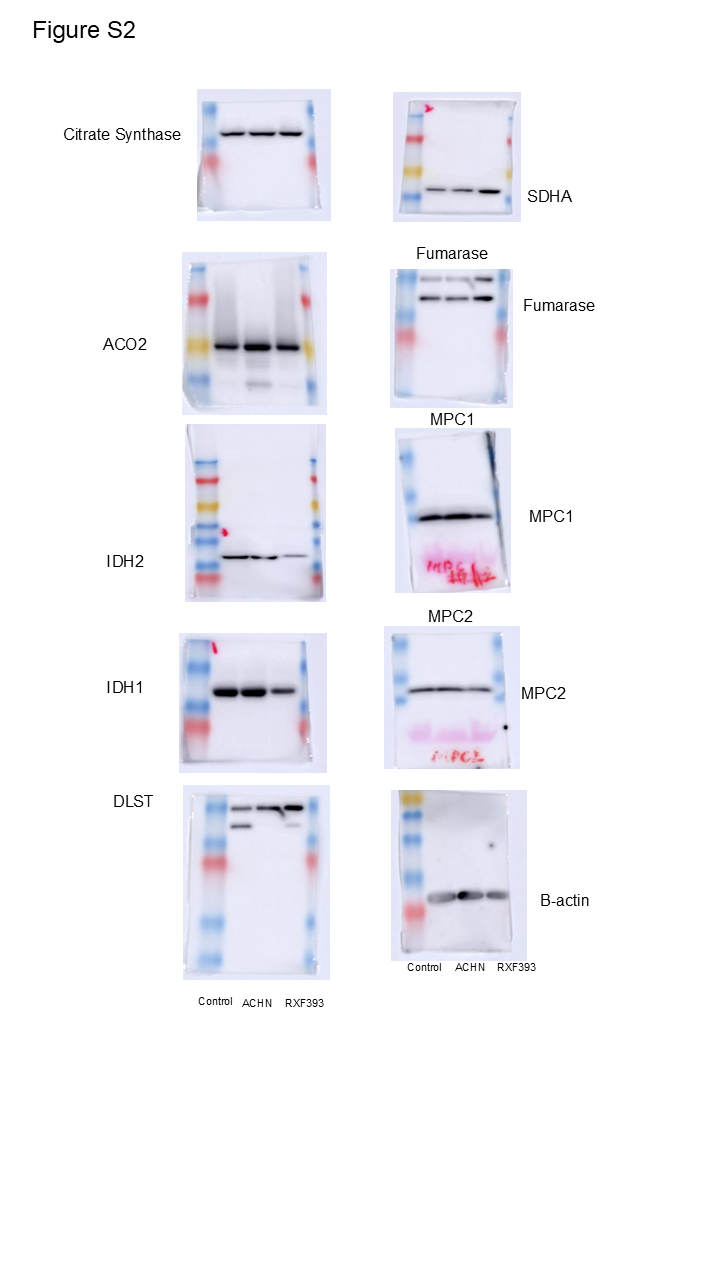

Supplement: Supplementary Figure 2 — Raw Western blot images correspond to Figure 2A. [file Image2.tif]

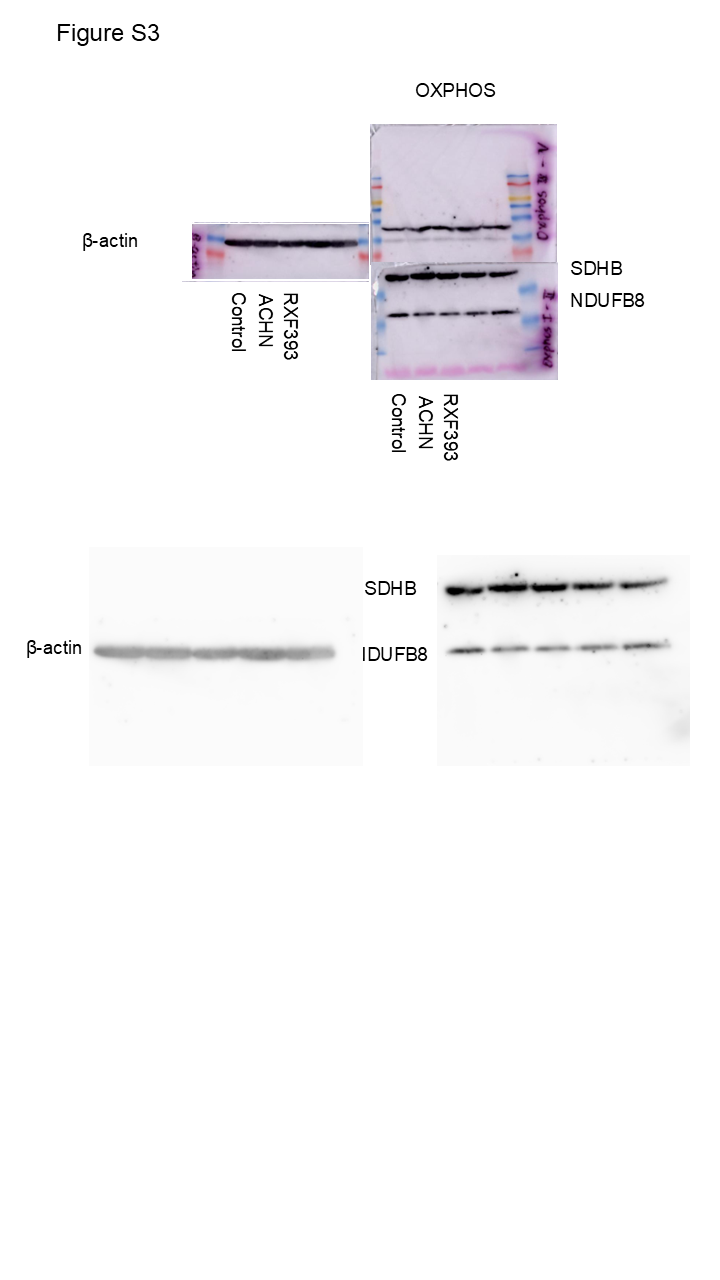

Supplement: Supplementary Figure 3 — Raw Western blot images correspond to Figure 2B. [file Image3.tif]

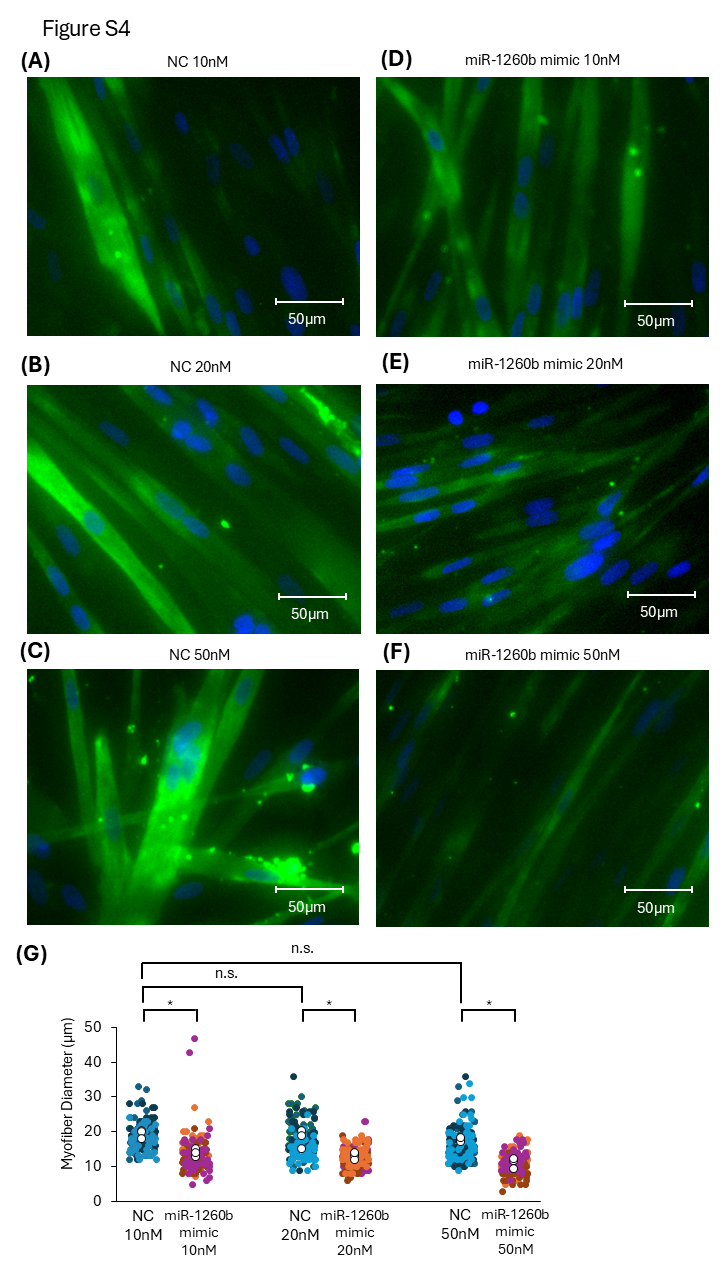

Supplement: Supplementary Figure 4 — Effects of miR-1260b mimic on myotube diameter in skeletal muscle cells. Skeletal muscle cells were transfected with miR-1260b mimic or negative control (NC) at the indicated concentrations (10, 20, and 50 nM). Representative images are shown for NC (A–C) and miR-1260b mimic (D–F). Quantification of myotube diameter is shown in (G). Data are presented as mean ± SEM (n = 3 independent experiments). Scale bar = 50 μm; *p < 0.05 by Student’s t-test.S [file Image4.tif]

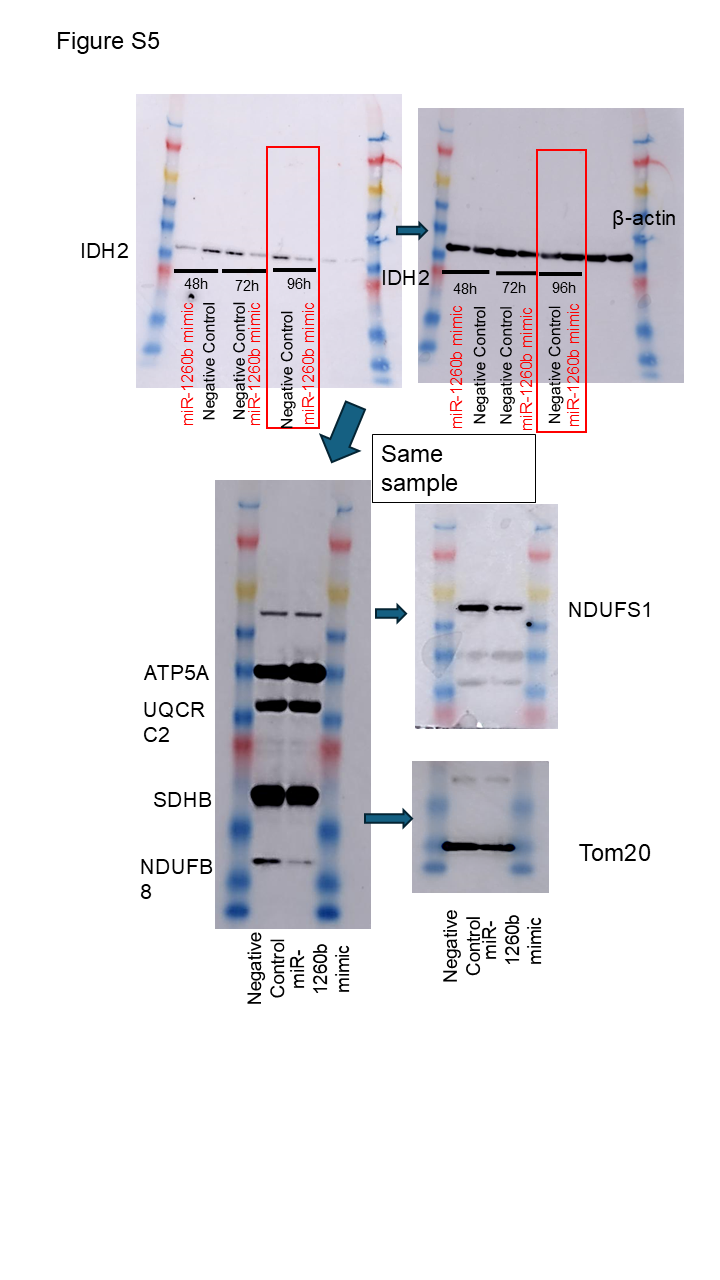

Supplement: Supplementary Figure 5 — Raw Western blot images correspond to Figure 3G. [file Image5.tif]
